# Essential Contribution of Macrophage Tie2 Signalling in a Murine Model of Laser-Induced Choroidal Neovascularization

**DOI:** 10.1038/s41598-020-66580-y

**Published:** 2020-06-15

**Authors:** Xue Yin, Bingyu Zhang, Lei Chen, Wei Xia, Gaoqin Liu, Xuefei Zhu, Chi Ren, Weiming Liu, Peirong Lu

**Affiliations:** Department of Ophthalmology, the First Affiliated Hospital of Soochow University, Suzhou, China

**Keywords:** Inflammation, Inflammation

## Abstract

Wet age-related macular degeneration (AMD), which can cause progressive blindness, is characterised by choroid neovascularization (CNV) in the macular area. Although close attention has been paid to AMD, and anti-vascular endothelial growth factor (VEGF) drugs are available, its complex pathogenesis is still elusive. Tie2-expressing macrophages (TEMs) have been found to promote angiogenesis in remodel tissues and tumours. This study aimed to elucidate the role of macrophage Tie2 signalling in laser-induced CNV (LCNV). We observed that TEMs were responsible for the severity of CNV. Mechanistically, TEM deletion resulted in impaired LCNV due to the suppression of inflammatory angiogenesis and the promotion of apoptosis. We also observed that TEMs prevented apoptosis of b.End3 cells, but promoted their migration, proliferation and tube formation via VEGF, extracellular signal-regulated kinase (ERK) and v-akt murine thymoma viral oncogene (AKT)-dependent signalling pathways. The flow cytometry results comparing dry AMD patients and healthy controls with wet AMD patients showed that the percentage of Tie2^+^CD14^+^ cells was higher in the wet AMD patients’ peripheral blood. This study demonstrates that Tie2 expression by macrophages intensifies CNV in LCNV murine models, thereby proposing an additional intervention option to inhibit CNV.

## Introduction

Choroidal neovascularization (CNV) is a terminal symptom of age-related macular degeneration (AMD), which is directly related to aging and chronic stress diseases^1^. Inflammation plays an important role in neovascular AMD (nvAMD). It was suggested that AMD is triggered by a chronic low-grade, whole body and local inflammatory response^2^. These immunity activations have been found to manifest as the activation of complement, mononuclear cell recruitment and macrophage descendants^3^. Inflammatory-related genes expressed in monocytes and peripheral blood mononuclear cells have been reported in nvAMD patients^4^. A significant number of macrophages have been detected in AMD in human eyes, and they modulated the formation of CNV in a laser-induced CNV (LCNV) murine model^5–7^.

Tie2-expressing macrophages (TEMs) are a subpopulation of macrophages. Their presence and phenotype have been confirmed in human blood^8^. TEMs have been found to promote angiogenesis in remodel tissues and tumours^9^. Deletion of TEMs was reported to inhibit angiogenesis in limb ischemia, hepatocellular carcinoma and tumour relapse^10–12^. Furthermore, researchers have reported that, potentially, increased recruitment of TEMs plays a role in enhanced neovascularization^13,14^. Macrophage Tie2-signal mediated-autophagy plays a critical role in LCNV^14^. However, the actual role of TEMs in AMD is still unclear. Therefore, the present study was designed to investigate whether the mechanism for TEMs contributes to LCNV as a model of AMD.

## Results

### Accumulation of intra-choroidal TEMs increased during LCNV

**Figure 1:**
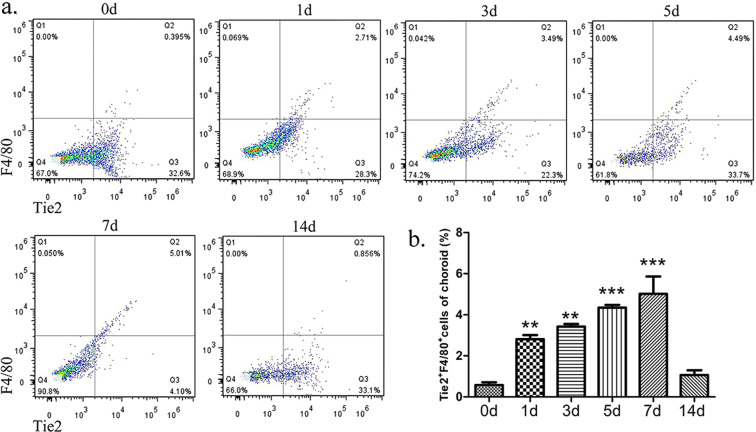
Time-dependent kinetic accumulation of intra-choroidal TEMs after laser injury. (**a**) The harvested choroids were analysed using flow cytometry at the indicated time points after laser injury. (**b**) The percentages of the choroidal infiltrating Tie2^+^F4/80^+^ cells were calculated at 0, 1, 3, 5, 7, and 14d. All values were recorded as mean ± SEM. In each of the six groups, *n* = 5 **p < 0.01. ***p < 0.001. Re*p*resentative results were obtained from three independent experiments.

To study the role of TEMs in LCNV, laser injury was induced to the choroid plexus of mice. We found that the injury promoted TEM accumulation. Single-cell suspensions were digested from the retinal pigment epithelium (RPE)-choroid tissue of the C57BL/6J mice. In the fluorescent-activated cell sorting (FACS) analysis, the time-dependent percentages of Tie2^+^/F4/80^+^ macrophages were 0.577 ± 0.131% at 0d, 2.813 ± 0.195% at 1d, 3.420 ± 0.129% at 3d, 4.340 ± 0.135% at 5d, 5.017 ± 0.849% at 7d and 1.06 ± 0.235% at 14d (mean ± SEM). The results showed that the TEMs infiltrated the choroid plexus within 1d after laser injury, and then gradually increased from 3d to 5d, peaking at 7d (Fig. 1a,b), which suggests that LCNV was closely associated with Tie2 signalling on macrophages. No significant difference in intra-choroidal F4/80^+^ cell infiltration was found between the TEM-knockout (TEM-KO) mice and the control mice (Supplementary Fig. 1), suggesting that macrophage Tie2-specific deletion had no effect on macrophage recruitment.

### Macrophage Tie2-deletion decreased LCNV

**Figure 2:**
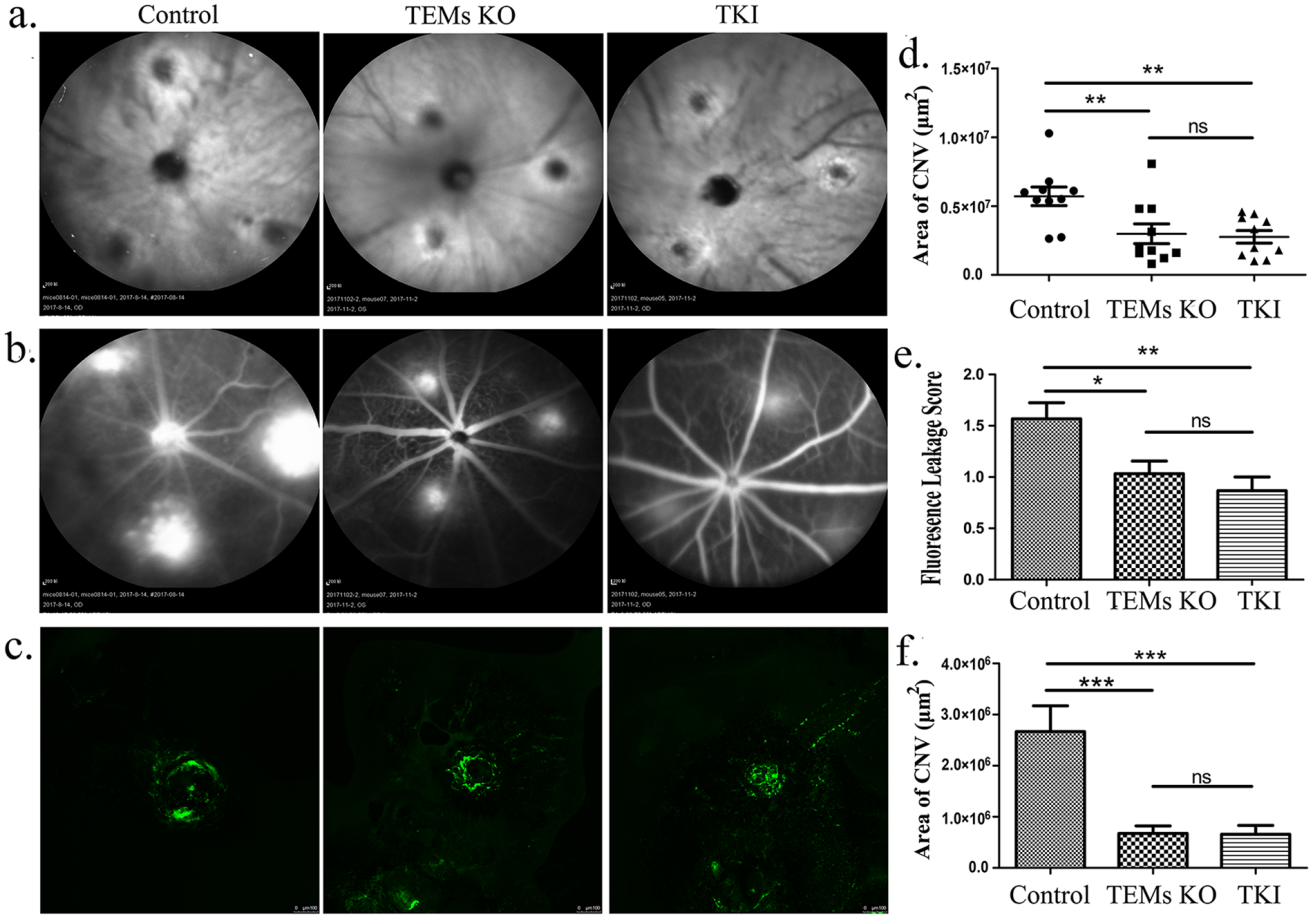
Laser-induced CNV. (**a,b**) CNV was induced in the control, TEM-KO and specific Tie2 kinase inhibitor (TKI)-injected mice. Fundus photographs were taken, and fundus fluorescein angiograms (FAAs) were obtained 7d after laser treatment. Scale bar: 200 μm. (**c**) The area of the neovascular complexes (green) was determined using confocal microscopy. Scale bar: 100 μm. (**d,e**) Using the FFAs, the CNV area and the CNV leakage scores were analysed using one-way ANOVA in the control, TEM-KO and TKI groups. (**f**) Using the choroid flat mounts with fluorescent dextran cardiac perfusion, differences in the CNV area in the groups were calculated using one-way ANOVA. All values were recorded as mean ± SEM. In each group in the FFA and flat mount analysis, n = 10. *p < 0.05. **p < 0.01. ***p < 0.001. Representative results were obtained from three independent experiments.

TEMs have been found to be beneficial for LCNV^14^. To further determine whether TEMs participate in LCNV, a Tie2 gene KO was induced on the macrophages using a Cre-loxP system, as described in the Materials and Methods section. We examined the CNV lesion area and assessed the CNV leakage score in the choroid plexus of the mice (Fig. 2a). Using fluorescein fundus angiogram (FFA), we obtained the following CNV area results (Fig. 2d): 5.70 ± 0.68 × 10^6^/μm^2^ (control group), 2.98 ± 0.72 × 10^6^/μm^2^ (TEM-KO group) and 2.76 ± 0.46 × 10^6^/μm^2^ (Tie2 kinase inhibitor [TKI] group) (mean ± SEM), respectively. In the TEM-KO and TKI groups, the CNV areas exhibited less fluorescence leakage 1 week after laser photocoagulation (Fig. 2b,e). As shown in the fluorescent dextran choroid flat mount (Fig. 2c), the CNV area of the irrigated area was significantly smaller in the TEM-KO group (0.67 ± 0.15 × 10^6^/μm^2^) and the TKI group (0.66 ± 0.17 × 10^6^/μm^2^) than the control group (2.66 ± 0.5 × 10^6^/μm^2^) (Fig. 2f). To confirm this result, we observed the effect of the TKI treatment on the laser-induced CNV, and we used anti-vascular endothelial growth factor (VEGF) (Lucentis, 1 μl, intravitreal injected 3 days before laser injury) as the control. The reproductive results showed that TKI played a protective role in LCNV (Supplementary Fig. 2). Moreover, this TKI agent showed no significant inhibition of VEGFR2 phosphorylation (Supplementary Fig. 3). These data, together with our previous results^14^, suggest that macrophage Tie2 deletion resulted in impaired LCNV. Furthermore, the suppression of CNV induced by TEMs was not less than the suppression induced by TKI.

### Angiogenic factor expression decreased and apoptotic maker expression increased in the TEM-KO group

**Figure 3:**
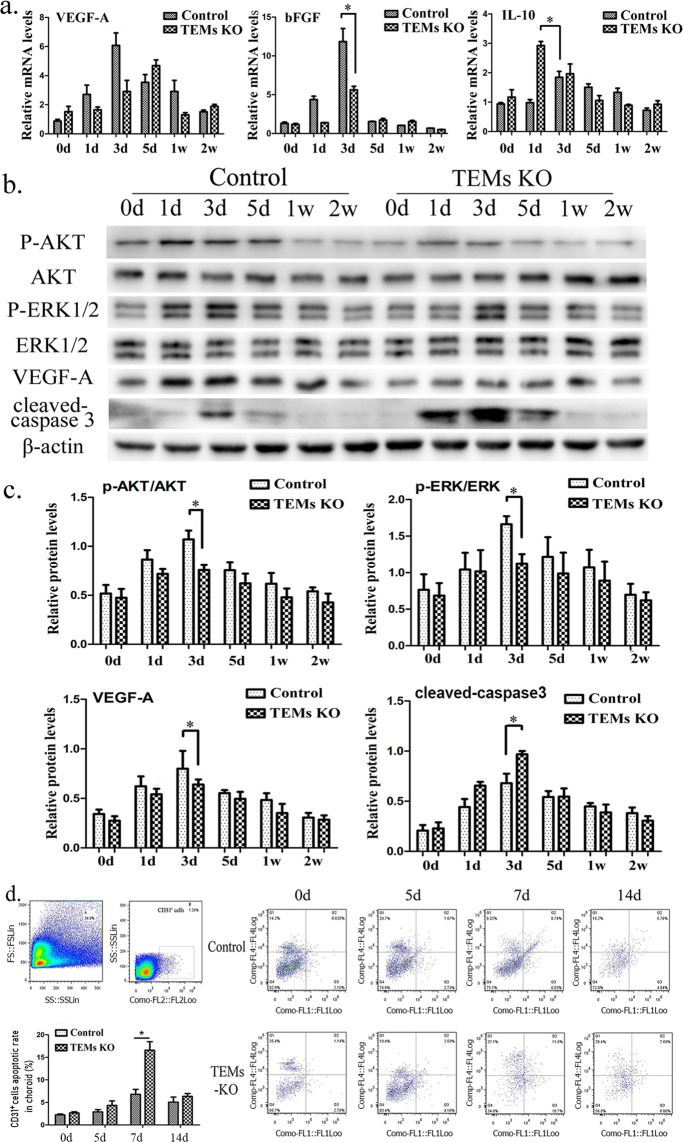
(**a**) Quantitative reverse transcription polymerase chain reaction (RT-PCR) to assess mRNA expression of VEGF-A, bFGF and IL-10 with the total RNAs obtained from the control/TEM-KO mice choroids at different time points (0, 1, 3, 5, 7, and 14d). (**b**) AKT, p-AKT, ERK, p-ERK, VEGF-A and cleaved caspase-3 protein expression was detected using Western blot in the injured choroid plexus from the control/TEM-KO mice. (**c**) The mean grey value of the Western blot was determined and described as mean ± SEM. Cropped blots are displayed in the figure, and full-length gels and blots were included in the Supplementary information file. (**d**) In the flow cytometry detection, the apoptotic rate of the CD31^+^ cells of the mice choroid were tested at 0, 5, 7, and 14d after laser injury. *N* = 30 (control group); *n* = 30 (TEM-KO group). The left superior two images represent the viable cells and the CD31^+^ cells among the viable cells. *p < 0.05. Representative results were obtained from three independent experiments.

Due to their critical roles in CNV formation, we measured the intra-choroidal expression levels of angiogenic factors and inflammatory cytokines during LCNV (Fig. 3a). In the mouse RPE-choroid, the mRNA expression of VEGF-A and bFGF gradually increased, and then it decreased over a period of 14d after the laser injury was induced. The peak expression levels of VEGF-A and bFGF were lower in the TEM-KO group (VEGF-A: 6.078 ± 0.856; bFGF: 11.840 ± 1.694) than the control group (VEGF-A: 4.690 ± 0.394; bFGF: 5.623 ± 0.461). Moreover, the expression level of VEGF-A in the TEM-KO group did not peak until 5d. At the same time, the peak value of interleukin-10 (IL-10) was higher and occurred earlier in the TEM-KO group (2.924 ± 0.140) than the control group (1.845 ± 0.205). Western blot analysis (Fig. 3b,c) also demonstrated that the protein expression level of VEGF-A peaked at 3d. The maximum value of VEGF-A was lower in the TEM-KO group (0.640 ± 0.051) than the control group (0.799 ± 0.180). The dynamic change of VEGF-A was consistent with our previous research finding^14^.

Macrophage depletion affected the expression levels of inflammation and the number of apoptosis genes, and it played a critical role in supporting the development of the vascular network^15^. Hence, we determined the protein expression of the angiogenic factors ERK and AKT and the apoptotic factor caspase-3 in the choroids after laser injury. The results showed that the peak expression levels of p-ERK and p-AKT were both substantially lower in the TEM-KO group (p-ERK: 1.122 ± 0.130; p-AKT: 0.760 ± 0.050) than the control group (p-ERK: 1.663 ± 0.110; p-AKT:1.070 ± 0.090). ERK and AKT phosphorylation promotes endothelial migration and proliferation, which has been shown to be a component of the VEGF signalling pathway^16^.

To investigate whether these effects were apoptosis-related, we monitored the apoptotic protein expression. After laser injury, the Western blot analysis results showed that the cleaved caspase-3 protein level at 3d was significantly higher in the TEM-KO group (0.968 ± 0.033) than the control group (0.680 ± 0.095). The apoptotic rates of the CD31^+^ cells were then detected using FACS. Single-cell suspensions were digested from the RPE-choroid tissue. The apoptotic rate was determined using annexin V and propidium iodide (PI) dual staining; this showed that the percentage of the peak value of the apoptotic CD31^+^ cells in the choroid plexus was higher in the TEM-KO group (16.570 ± 1.906%) than the control group (6.797 ± 1.091%) 7d after laser injury (Fig. 3d). Thus, it can be concluded that TEM-KO was responsible for vascular endothelium cell apoptosis in LCNV.

### TEM supernatant changed the expression of angiogenic factors in b.End3 cells, but it did not change macrophage polarization

**Figure 4:**
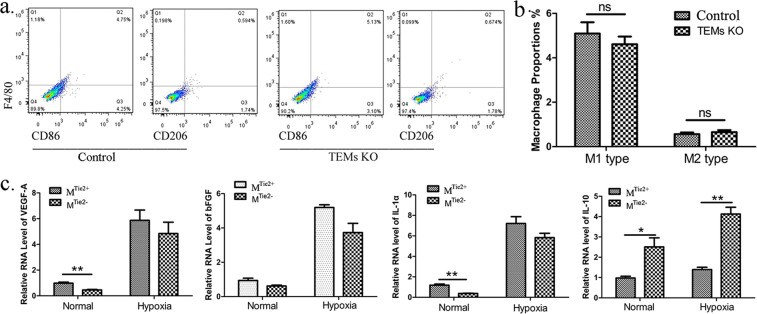
Macrophage polarization and its effect on vascular endothelial cells. (**a**) Polarization in the peritoneal macrophages. Peritoneal macrophages were obtained as described in the Materials and Methods section. The CD86^+^F4/80^+^ and CD206^+^F4/80^+^ cells from the control or TEM-KO mice were analysed using flow cytometer. (**b**) Histogram of the percentages of CD86^+^F4/80^+^ and CD206^+^F4/80^+^ cells. Statistical analysis was performed using the unpaired Student *t*-test. (**c**) Quantitative RT-PCR was performed to analyse the proangiogenic and antiangiogenic RNA expression in the b.End3 cells. The b.End3 cells were incubated under normal and hypoxia conditions with macrophage supernatants from the control and TEM-KO mice, as described in the Materials and Methods section. M^Tie2−^ group: b.End3 incubated with macrophage supernatant from the TEM-KO mice. M^Tie2+^ group: b.End3 incubated with macrophage supernatant from the control mice. *p < 0.05, **p < 0.01. Re*p*resentative results were obtained from three independent experiments.

It has been reported that Tie2 expression is not restricted to macrophages, and that Tie2 signalling is independent from the macrophage functional phenotype^17^. According to a previous study^18^, macrophages are activated and then polarized into M1 macrophages (CD86^+^F4/80^+^ cells) and M2 macrophages (CD206^+^F4/80^+^ cells). As expected, in our study, there was no significant difference between the percentages of M1 and M2 intraperitoneal macrophages in the TEM-KO group (M1: 4.613 ± 0.345%; M2: 0.655 ± 0.087%) and those in the control group (M1: 5.090 ± 0.514%; M2: 0.562 ± 0.076%) (Fig. 4a,b). This suggests that macrophage Tie2 signalling had no effect on macrophage polarization.

Hypoxia (1.5% O_2_) is one of the most important factors associated with cell damage in AMD^19^. VEGF and bFGF are both hypoxia-related cell factors that are involved in nvAMD pathology. These factors have been detected in the endothelium and macrophages of human choroidal neovascular membranes^20^. It has not yet been proven if this also applies to TEMs. To further verify the effect of TEMs on vascular endothelial cells, the b.End3 cells were cultured with macrophage supernatant under normal and hypoxic conditions. In both conditions, the mRNA levels of VEGF-A, bFGF, Interleukin 1 alpha (IL-1α) and IL-10 increased after macrophage supernatant incubation. Under the normal condition, the expression levels of VEGF-A and IL-1α were lower in the M^Tie2−^ group (VEGF-A: 0.455 ± 0.043; IL-1α: 0.383 ± 0.017) than the M^Tie2+^ group (VEGF-A: 0.986 ± 0.079; IL-1α: 1.191 ± 0.106), but the IL-10 expression level was higher in the M^Tie2−^ group (M^Tie2−^: 1.395 ± 0.113; M^Tie2+^: 0.976 ± 0.089). The IL-10 expression level was also higher in the M^Tie2−^ group (M^Tie2−^: 4.126 ± 0.333; M^Tie2+^:2.511 ± 0.446) under the hypoxia condition (Fig. 4c). Hypoxia dysregulated the expression of these angiogenesis-related factors. Tie2 deficiency did not reverse the mRNA expression trends in the M^Tie2+^ and M^Tie2−^ groups under the hypoxic condition.

### TEM supernatant inhibited the migration and proliferation of b.End3 cells, but it increased their apoptosis ability

**Figure 5:**
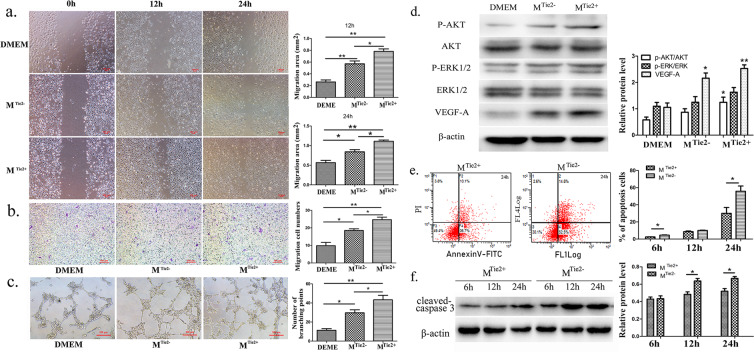
The effects of the TEM supernatant on the b.End3 cells. The b.End3 cells were incubated with DMEM, macrophage supernatant from the control mice (M^Tie2+^) and macrophage supernatant from the TEM-KO mice (M^Tie2−^), respectively. (**a**) In the scratch wound assay, the b.End3 cells were treated with the macrophage supernatant for 12 h and 24 h. Statistical analysis was performed using one-way ANOVA. Scale bar: 100 μm. (**b**) In the transwell migration assay, the b.End3 cells in the different study groups were incubated for 24 h. Scale bar: 100 μm. (**c**) In the tube formation assay, the b.End3 cells in the different study groups were incubated for 6 h. Scale bar: 200 μm. (**d**) Representative results of p-AKT, AKT, p-ERK, ERK and VEGF-A are presented, as determined by Western blot, from three independent experiments. (**e**) In the annexin V/PI dual-staining flow cytometry detection, the apoptosis rate of the b.End3 cells was tested after 6, 12, and 24 h incubation with the macrophage supernatant. (**f**) Representative results, as determined by Western blot, from three independent experiments are presented. The relative cleaved caspase-3 protein expression of the b.End3 cells under different macrophage supernatants is presented. Cropped blots are displayed in the figure, and full-length gels and blots were included in Supplementary Information file. All values represent mean ± SEM. *p < 0.05, **p < 0.01. Representative results were obtained from three independent experiments.

It has been reported that TEMs promote the migration and proliferation of b.End3 cells^14^. To confirm the migration and proliferation effect of TEMs on endothelial cells, murine peritoneal macrophages were isolated and macrophage supernatant was collected to culture b.End3 cells. The wound assay results (Fig. 5a) indicate that the supernatants promoted the migration and proliferation of b.End3 cells more than the Dulbecco’s modified Eagle’s medium (DMEM) group at both 12 h (DMEM 0.262 ± 0.034 mm^2^; M^Tie2−^: 0.571 ± 0.048 mm^2^, M^Tie2+^: 0.782 ± 0.042 mm^2^) and 24 h (DMEM: 0.564 ± 0.059 mm^2^, M^Tie2−^: 0.842 ± 0.055 mm^2^, M^Tie2+^: 1.111 ± 0.031 mm^2^). Moreover, the migration and proliferation of b.End3 cells were significantly greater in the M^Tie2+^ group than the M^Tie2−^ group. The results of a transwell migration assay (Fig. 5b) showed that the increase in the number of migrated cells was greater in both the M^Tie2+^ group (24.670 ± 1.453) and the M^Tie2−^ group (18.500 ± 0.957) than in the DMEM group (9.833 ± 1.869). Cell migration was also promoted to a greater extent in the M^Tie2+^ group than in the M^Tie2−^ group. As shown in Fig. 5c, a tube formation assay revealed that, under different stimulated conditions, the number of tube nodes was higher in the M^Tie2+^ group (43.330 ± 4.631) than the M^Tie2−^ group (29.670 ± 3.180) and the DMEM group (11.330 ± 1.764).

As shown in Fig. 5d, the Western blot analysis results revealed that the protein expression level of VEGF-A was significantly higher in the M^Tie2+^ group (2.529 ± 0.138) and the M^Tie2−^ group (1.628 ± 0.178) than in the DMEM group (1.247 ± 0.193). We also reproduced these results using another mouse vascular endothelial cell: C166. The results, shown in Supplementary Fig. 3, indicate that VEGF-A expression in C166 after stimulation was consistent with that in b.End3 (DEMD: 0.038 ± 0.086; M^Tie2−^: 0.518 ± 0.030; M^Tie2+^: 0.844 ± 0.137) (Supplementary Fig. 4). The relative expression level of p-AKT was higher in the M^Tie2+^ group (1.053 ± 0.169) than the M^Tie2−^ group (1.095 ± 0.145) and the DMEM group (0.579 ± 0.109). However, there was only an increasing tendency; no statistically significant increase was seen in the relative expression level of p-ERK in the macrophage supernatant–incubated group in comparison to the DMEM group.

Time-dependent kinetics of b.End3 apoptosis on annexin V/PI dual staining demonstrated that exposure to the macrophage supernatant increased b.End3 apoptosis. The number of annexin V^+^PI^−^ cells significantly increased in both the M^Tie2−^ and M^Tie2+^ groups at 24 h (Fig. 5e). The apoptotic rate of the b.End3 cells was significantly higher in the M^Tie2−^ group (4.587 ± 0.286% at 6 h, 55.800 ± 6.088% at 24 h) than in the M^Tie2+^ group (2.490 ± 0.144% in 6 h, 30.030 ± 6.868% at 24 h) after macrophage supernatant incubation. Similarly, the relative cleaved caspase-3 protein expression level of b.End3 gradually increased in both the M^Tie2+^ and M^Tie2−^ groups after incubation in the macrophage supernatant for 6 h, 12 h and 24 h (Fig. 5f). Moreover, it was significantly higher in the M^Tie2−^ group than in the M^Tie2+^ group after incubation for 12 h and 24 h. This suggests that the deletion of TEMs activated b.End3 apoptosis.

### Peripheral blood Tie2-associated monocytes (TAMs) increased in wet AMD patients

**Figure 6:**
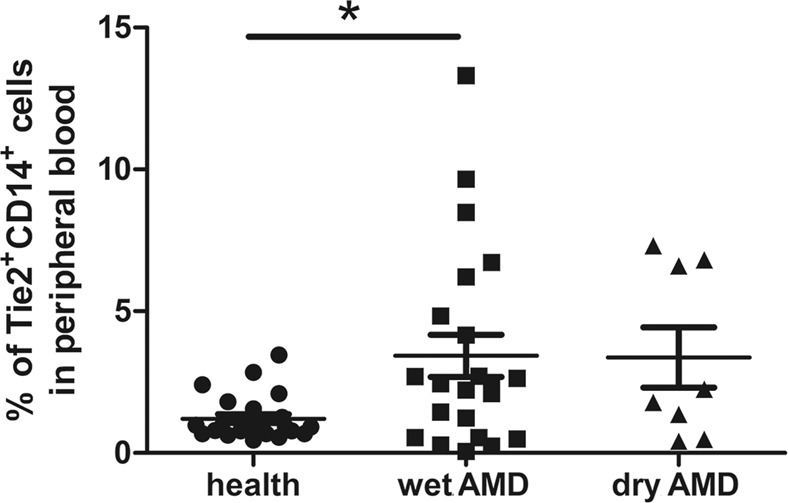
The percentage of Tie2^+^CD14^+^ cells in human peripheral blood. Blood samples were obtained from 22 wet AMD patients, 8 dry AMD patients and 24 healthy controls, and they were evaluated using a flow cytometer. *P < 0.05, wet AMD patients versus healthy controls using post-hoc test in one-way ANOVA.

Since it is almost impossible to obtain human choroid tissue in a clinical setting, we attempted to detect the expression level of TEMs in peripheral blood. However, macrophages are difficult to detect in peripheral blood. It has been reported that monocytes in peripheral blood can infiltrate various target organs and differentiate into macrophages^21^. Thus, we determined the percentages of Tie2^+^CD14^+^ cells in the peripheral blood of AMD patients and healthy (control) subjects. Thirty AMD patients (22 wet AMD and 8 dry AMD) and 24 age-matched control subjects were recruited for this part of the study. As detected by FACS (Fig. 6), no statistically significant difference in the percentage of Tie2^+^CD14^+^ cells was observed between the dry AMD patients (3.368 ± 1.057%) and the healthy subjects (1.209 ± 0.158%); however, the percentage of Tie2^+^CD14^+^ cells was significantly higher in the wet AMD patients (3.426 ± 0.743%) than the healthy age-matched controls.

## Discussion

Currently, the primary treatment for wet AMD targets VEGF^22,23^. However, in addition to the economic burden of this strategy, anti-VEGF therapy resistance poses a significant treatment challenge, underlining the need to identify a new, more efficient therapy. In this degenerative disease, deposits within the lesion contain many inflammatory mediators^24^. Furthermore, AMD is associated with a local inflammatory response and systemic activation of innate immunity. This inflammatory response includes the activation of monocytes and descendant macrophages^3,25^. Anti-inflammatory therapy is another potential option for treating AMD.

Traditionally, TEMs have been considered to accumulate in the tumour microenvironment, especially after chemotherapy or radiotherapy^26^. They have also been found to increase in limb ischemia after muscle injury^10^. They play an important role in revascularization, which is associated with angiogenesis and vascular reconstruction^27,28^. In this study, we found that laser injury promoted Tie2-expressing F4/80-positive macrophage intra-choroidal infiltration. Thus, we speculated that TEMs are involved in CNV. To confirm this hypothesis, we investigated the function of TEMs using an LCNV murine model. The CNV area was impaired in the TEM-KO mice, indicating that TEMs might be a contributing factor for LCNV, and TEM deletion was found to be beneficial for inhibiting CNV. The invasiveness of TEMs in the choroid plexus following laser injury may represent a kind of pro-inflammatory response, which can provide a new target for overcoming CNV in a clinical setting. Moreover, the inhibition effect induced by TEMs was not weaker than that induced by TKI. Thus, our results support the supposition that TEMs might be an effective and potential target in wet AMD.

In LCNV in mice, overexpression of Ang1 was found to suppress CNV formation and vascular leakage by inhibiting the recruitment of angiogenic macrophages and tightening the endothelial junctions^29^. Tie2 activation by Ang 2 binding and Tie2-activating antibody (ABTAA) was also reported to alleviate CNV and vascular leakage^30^. These findings indicate that Tie2 activation has a strong protective effect in an LCNV model. However, Hangai reported that systemically expressed soluble Tie2 by adenovirus-mediated gene delivery of extracellular domain of the Tie2 receptor, which suppressed endogenous Tie2, could inhibit experimental retinal and choroidal neovascularization^31^. Semones observed that Tie2-specific kinase inhibitor treatment led to a reduction in bFGF-induced sprouting angiogenesis and impaired tumour growth in a xenograft model^32^. These data support our finding that global Tie2 inhibition by TKI in the eye suppresses LCNV and replicates the phenotype of macrophage-specific. It is worth noting that TKI was used in this study as a control; to rule out the possibility that TKI treatment results in VEGF receptor inhibition, we detected the receptor 2 and phosphorylated receptor 2 of VEGF after TKI treatment. We found no considerable inhibition of VEGFR2. At present, we cannot explain these seemingly contradictory findings, and more investigative work is needed to explore the mechanism of Tie2 signalling on LCNV.

Although our results illustrate the mechanism whereby CNV formation contributes to TEMs, it is important to investigate how TEMs inhibit CNV after laser injury. Angiogenic factors have been reported to be implicated in the pathogenesis of AMD^33^. In neovascularization, macrophages infiltrating from myeloid lineage are a key source of the angiogenic factors^34^. Tie2 signalling especially plays an important role in the regulation of the expression of pro-inflammatory cytokines and chemokines in macrophages^35^. MRC1^+^TIE2^Hi^CXCR4^Hi^ macrophages accumulate and promote tumour revascularization partly via VEGF-A release. The factors expressed by human TEMs include VEGF-A, MMP-9 and IL-10, whose expression is important for angiogenesis and immune regulation^36,37^. Similarly, we found that the pro-angiogenic effect of TEMs during CNV might be correlated with VEGF-A, bFGF and IL-10 secretion. The mechanism of neovascularization after laser injury might be associated with these TEM-related pro-angiogenic factors. Moreover, the deletion of Tie2 did not alter the percentages of M1 or M2 in the peritoneal macrophages of the TEM-KO mice in comparison to the control mice. Although Tie2 expression has been observed under all macrophage polarization conditions^17^, it is not associated from macrophage functional classification.

In addition to the previously-mentioned observations about the deletion of TEM, we investigated how the TEM-related proangiogenic factors were secreted. We found that the increase in the mRNA levels of VEGF-A, bFGF, IL-1α and IL-10 in the M Tie2+ and M Tie2− supernatant-cultured b.End3 cells tended to be greater under the hypoxic condition than the normal condition. A previous study reported that vascular endothelial cells expressed VEGF-A under certain stimuli^38^, and that high amounts of VEGF-A released by macrophages promoted neovascularization^39^. Hypoxia has been reported to up-regulate the cell surface expression of Tie2 on human macrophages and monocytes^13,40^. To prevent the effect of hypoxia on TEMs, we stimulated b.End3 in a hypoxic condition with macrophage supernatants rather than with a cell co-culture. Our observations suggest that angiogenic factors were secreted by the combined effect of hypoxia and TEMs in b.End3. Unfortunately, the mechanism of how the secretion of angiogenic factors is initiated by Tie2 signalling on macrophages is still unknown. In the future, additional studies should be conducted to investigate the composition of the macrophage supernatants.

From a pathophysiological point of view, the Tie2 signal was closely associated with macrophage apoptosis, and the apoptosis was induced via the AKT-dependent signalling pathway^13^. Furthermore, in a cerebrovascular rupture animal model, Liu reported that macrophages could generate mechanical traction forces and pull endothelial ends to facilitate their ligation^41^. Obviously, there is a close connection between macrophages and vascular endothelial cells. However, no previous study has reported on how TEMs effect b.End3 cells in LCNV. In this study, the Western blot assay results demonstrated the augmented expression of cleaved caspase-3, a protein marker of apoptosis, in the TEM-KO mice. A similar outcome was detected using annexin V/PI flow cytometry; the apoptosis rate of the b.End3 cells increased after macrophage supernatant incubation, and the apoptosis rate was more obviously augmented in the M^Tie2−^ group than in the M^Tie2+^ group. In fact, deletion of TEMs suppressed the p-AKT and p-ERK expression levels; this suggests that TEMs might be beneficial for b.End3 cell proliferation and migration. Logically, it was then possible to perceive that TEMs inhibited b.End3 apoptosis, and the process might occur via ERK and the AKT-dependent signalling pathway.

In healthy individuals, TAMs account for 2–7% of the mononuclear cells in blood^42^. A previous study revealed that TAMs are beneficial for angiogenesis in peripheral blood^43^. Monocytes in peripheral blood can infiltrate into various tissues, and they mainly differentiate into macrophages^21^. Using FACS analysis, the present study found that the number of Tie2^+^CD14^+^ cells in human peripheral blood significantly increased in the wet AMD patients, which suggests that the number of TAMs in peripheral blood increased in the wet AMD patients. Moreover, some researchers have suggested that monocytes can replace resident macrophages in organs and adopt macrophage tissue-specific gene expression^43,44^. Based on the present study’s observations, it is postulated that peripheral blood TAMs migrate into CNV lesions and differentiate into TEMs in wet AMD patients, which, subsequently, intensifies vascular leakage and neovascularization. Interestingly, in anti-VEGF-treated tumour patients, TEMs were found to be triggered by anti-VEGF therapy^45^. Therefore, it is reasonable to believe that TEMs could be an adequate factor for AMD development, even for anti-VEGF-resistant wet AMD patients.

In conclusion, this study found that macrophage Tie2 signalling can affect vascular endothelial cell tube formation through its own secreted pro-angiogenic factors and through its angiogenic and apoptotic mechanisms on endothelial cells, thereby significantly regulating neovascularization. Re-education of macrophages, especially targeting Tie2 signalling, is a potential intervention method for wet AMD.

## Materials and Methods

The study was approved by the Ethics Review Board of the First Affiliated Hospital of Soochow University. We abided by the tenets of the Declaration of Helsinki, and we registered this study in the Chinese Clinical Trial Registry as ChiCTR-RPC-17011329. All the human subjects that participated in this project signed an informed consent form. All the animal experiments followed the Guidelines for the Care and Use of Laboratory Animals of the Chinese Medical Academy and were approved by the Soochow University Animal Care Committee (Suzhou, China); moreover, they were performed in accordance with the ARVO Statement for the Use of Animals in Ophthalmic and Vision Research.

### Cell cultures

Mouse brain arteriole vessel endothelium cells (b.End3) were cultured in Dulbecco’s modified Eagle’s medium (DMEM) (Gibco, Grand Island, NY, USA) with 10% foetal bovine serum (FBS) (Gibco) at 37 °C in a humidified atmosphere (5% CO_2_, 95% air). The cells were plated at an appropriate density according to the experimental design.

Then, 2 mL sterile 3% thioglycolate medium (Sigma-Aldrich, St. Louis, MO, USA) was injected intraperitoneally. Three days later, the macrophages were harvested with 5 mL of phosphate buffered saline (PBS) by peritoneal lavage, as previously described^46,47^. A quantity of 1 × 10^6^ cells were counted and seeded in a T25 cell culture flask. Nonadherent cells were removed 2 h later, and the macrophages were cultured for another 24 h. Macrophage supernatants were then collected. The proportion of F4/80^+^ macrophages was >95% in all the resultant cells, which was verified by flow cytometry analysis (Supplementary Fig. 5). TEM-KO was verified by Western blot assay (Supplementary Fig. 6).

### Animals

The generation of Tie2^flox/flox^ mice was based on the background of C57BL/6J mice. LyzCre^+/+^ and Tie2^flox/flox^ mice were bred to produce LyzCre^+^; Tie2^flox/+^ mice. LyzCre^+^; Tie2^flox /+^ mice (control) and LyzCre^+^; Tie2^flox /flox^ mice (TEM-KO group) were produced by mating the LyzCre^+^; Tie2^flox/+^ mice with the Tie2^flox/flox^ mice^13^. All the animal studies were performed with 8- to 12-week-old sex-matched mice.

### LCNV model

To mimic the CNV of wet AMD, spots were placed on the peripapillary avascular zone with a 532 nm Nd: YAG Laser (240 ms, 50 µm spot size, three spots per eye). Using a microscopic delivery system, a laser burn was placed three times around the optic disc.

### Intravitreal injection of CAS948557-43-5

A specific Tie2 kinase inhibitor (TKI), CAS948557-43-5 (Merck & Millipore, Darmstadt, Germany), was dissolved with dimethylsulphoxide (DMSO) in the following concentrations: 0.01 mmol/L, 0.1 mmol/L, 1.0 mmol/L and 10.0 mmol/L. Then, 1 μl dilution was intravitreal injected with a 33 G blunt needle (World Precision Instruments Inc., Florida, USA) 3 days before the laser burns were placed around the optic disc. After comparing the effects and complications in the different dose groups, the 1.0 mmol/L concentration was selected in this study, and DMSO was set as the control.

### Fluorescein sodium angiography

To evaluate the area of the CNV lesion, the mice were anesthetised with Avertin (0.2 ml/10 g; Sigma-Aldrich, St. Louis, MO, USA) and injected with fluorescein sodium, intraperitoneally (60 mg/kg; Akorn, Copiague, NY, USA). Photographs and angiograms were taken with a TRC-50IA retinal camera (Topcon, Tokyo, Japan) and evaluated using Image J 1.46 software (US National Institutes of Health) by two blinded readers. The area of LCNV was defined as the average of three CNV lesions.

### Choroid flat mounts

The mice were euthanized 1 week (7d) after laser photocoagulation. The eyes were extracted after 20 mg/ml fluorescent dextran cardiac perfusion. Retinal pigmental epithelium (RPE) complexes (RPE/choroid/sclera) were radial cut and prepared for flat mounts. CNVs were viewed using confocal microscopy (LSM710, Carl Zeiss, Jena, Germany). The CNV area was measured using Image J software.

### Scratch wound assay

The b.End3 cells were starved for 24 h, and then seeded in 6-well plates with a density of 1.5 × 10^4^ per well. A sterile 200 μl pipette tip was used to scrape the monolayer cells. After rinsing the detached cells, 2 ml of supernatant was added to each well. The M^Tie2−^ group consisted of b.End3 cells incubated with macrophage supernatant from the TEM-KO mice. The M^Tie2+^ group consisted of b.End3 cells incubated with macrophage supernatant from the control mice. The DMEM group consisted of b.End3 cells cultured with DMEM. Photos of the wounded area were taken immediately at the time of wounding and at 12 h and 24 h, using an inverted microscope digital camera system (Olympus, Tokyo, Japan). The cell migration area was measured in five random microscopic fields using Image J software.

### Transwell migration assay

Transwell migration assays were performed using transwell filters (8 µm pore size, 24 wells, Corning, UK). The b.End3 cells were starved for 24 h. The 1.2 × 10^4^ cells were counted using a Neubauer haemocytometer, then suspended in 150 µl of DMEM medium (FBS-free), and then seeded into the upper chamber. Next, 600 µl of supernatants of peritoneal macrophages were added to the lower chamber. After 24 h incubation, the cells were fixed with methanol for 20 min and stained with 0.2% crystal violet. The number of coloured cells was measured using an inverted microscope in five random fields (Olympus, Tokyo, Japan).

### Tube formation assay

After being starved for 24 h, the b.End3 cells were cultured with supernatants of the control or the TEM-KO mice peritoneal macrophages for 24 h. Then, the b.End3 cells were trypsinized and cultured in 96-well plates (1.5 × 10^4^ cells/well) coated with 60 µl Matrigel matrix (cat. no. 356234; BD Biosciences, Franklin Lakes, NJ, USA). The tube branching point was quantified after 6 h by measuring the mean number in five random microscopic fields. The experiment was repeated three independent times.

### Real-time polymerase chain reaction (RT-PCR)

The b.End3 and mice choroids were harvested at various time points, according to the experiment design. Each choroid mRNA sample was extracted from 10 eyeballs. TRIzol reagent (Invitrogen, Grand Island, NY, USA) was used to extract the total RNA, following the manufacturer’s recommendations. Quantitative PCR reaction was performed with the following cycle conditions: 95 °C × 1 min, then 40 cycles of denaturation at 95 °C × 5 sec, and annealing/extension at 60 °C × 30 sec with the SYBR Green PCR Master Mix (Applied Biosystems, Warrington, UK). The RT-PCR primer pair sequences are listed in Supplementary Table 1. The relative mRNA level of the target gene was normalised to the levels of β-actin, and calculated using the 2 − ΔΔCt method.

### Western blot analysis

The target cells and mice choroids were harvested at various time points according to the experiment design. Each choroid protein sample in the different groups was extracted from 10 eyeballs. First, the antibodies specific for VEGF-A, total ERK 1/2, phosph-ERK 1/2, AKT, phosph-AKT and cleaved caspase-3 and β-actin (Cell Signaling Technology, Inc., Boston, MA, USA) were incubated at 4 °C overnight with dilutions of 1:1000. The polyvinylidene fluoride (PVDF) membranes were washed with a mixture of tris-buffered saline and Polysorbate 20 (TBST), three times. Then, the membranes were incubated with horseradish peroxidase-conjugated goat anti-mouse or anti-rabbit IgG (1:5000) for an additional 1 h. Finally, enhanced chemiluminescence was used to detect the immune complexes. All data were repeated at least three times.

### Flow cytometry

Annexin V/PI dual-staining was used to detect cell apoptosis. The b.End3 cells were plated in 60-mm wells (1 × 10^6^ cells/well) and incubated for 24 h with 4 mL of supernatants of the peritoneal macrophages from the control group (M^Tie2+^ group) and TEM-KO mice group (M^Tie2−^ group), respectively. Then, the adherent cells were collected and incubated with Annexin V/PI double-staining dye, according to the manufacturer’s protocol (BD Biosciences, San Diego, CA, USA). The samples were detected using Beckman Coulter FC500 Flow Cytometry (Beckman Coulter, Fullerton, CA, USA).

The RPE-choroid tissue was cut into small pieces and soaked in collagenase D solution for 45 min at 37 °C. Single-cell suspensions were digested from the tissue with 0.5 mg/ml collagenase D (20 units/ml; Roche Diagnostics, Mannheim, Germany). The TEMs were stained with fluorescein isothiocyanate (FITC)-conjugated anti-mTie2 and phycoerythrin (PE)-labelled anti-F4/80 (BD Biosciences). Apoptotic vascular endothelial cells were stained with PE-labelled anti-CD31 (BD Biosciences), Annexin V, and PI.

To detect the percentage of Tie2^+^CD14^+^ cells, 50 μl of human peripheral blood was collected and stained with FITC-labelled anti-CD14 and PE-labelled anti-hTie2. Then, the blood sample was lysed in 200 μl OptiLyse C (Beckman Coulter) for 15 min at 37 °C. Fluorescence intensities were detected using Beckman Coulter FC500 Flow Cytometry. Data were analysed using Flow Jo software (Tree Star, Ashland, OR, USA).

### Statistical analysis

Statistical analysis was performed using Predictive Analytics Software (PASW) Statistics 18 (IBM, NY, USA). All data in this experiment were expressed as means and standard errors of the means (SEM). Differences between the groups were compared with a two-tailed unpaired Student’s *t*-test or one-way analysis of variance (ANOVA). A post-hoc test was used for the correction of multiple comparisons. A value of *P* < 0.05 was considered statistically significant.

## Supplementary information

Supplementary information.

## Data Availability

The results of our analyses and the original images are available on request from the corresponding author.
