# Supplementary material for: Essential Contribution of Macrophage Tie2 Signalling in a Murine Model of Laser-Induced Choroidal Neovascularization
Source: Sci Rep. 2020 Jun 15;10:9613. doi: 10.1038/s41598-020-66580-y (PMC7295776; doi:10.1038/s41598-020-66580-y)
Supplement: Supplementary file 1 — Supplementary information. [file 41598_2020_66580_MOESM1_ESM.pdf]

Essential Contribution of Macrophage Tie2 Signalling in a Murine Model of Laser-Induced Choroidal Neovascularization

Xue Yin, Bingyu Zhang, Lei Chen, Wei Xia, Gaoqin Liu, Xuefei Zhu, Chi Ren, Weiming Liu, Peirong Lu

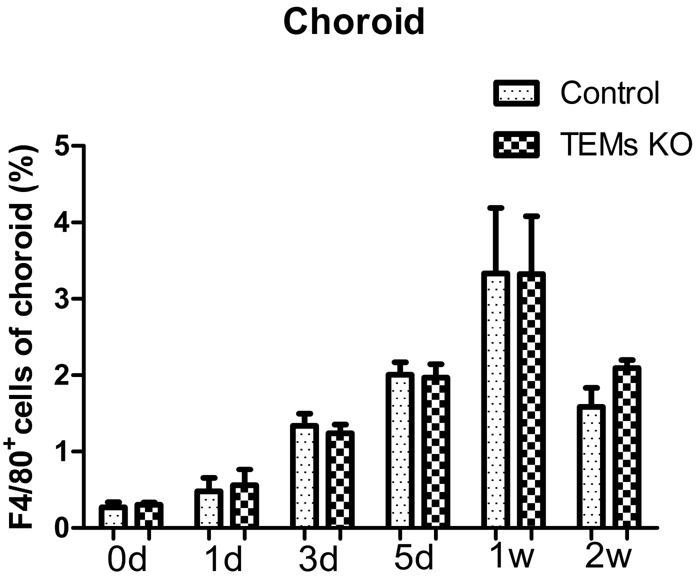

Supplementary Figure 1. Time-dependent kinetic accumulation of intra-choroidal TEMs after laser injury. The harvested single-cell suspensions were digested from choroids and analyzed using flow cytometry at the indicated time points after laser injury. The percentage of choroidal infiltrating F4/80$^+$ cells was calculated on 0d, 1d, 3d, 5d, 1w, and 2w, respectively.

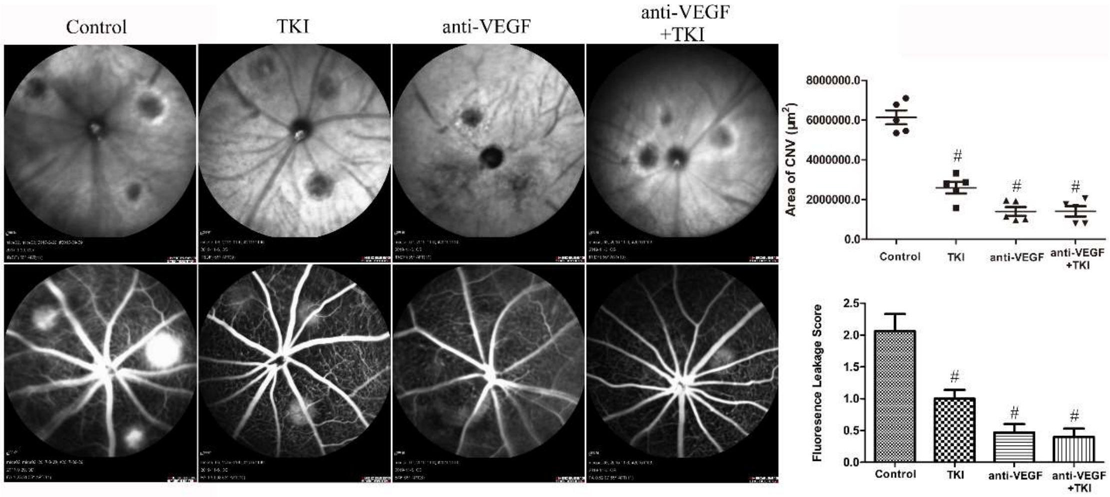

Supplementary Figure 2. Laser-induced CNV. CNV was induced in the control, TKI intravitreal injection mice (TKI group), anti-VEGF agent intravitreal injection mice (anti-VEGF group) and TKI + anti-VEGF group. Fundus photographs were taken and fundus fluorescein angiograms (FAAs) were obtained 7 d after laser treatment. Scale bar: 200μm. n =10 for each group. # $p< 0.001$. Representative results were obtained from three independent experiments.

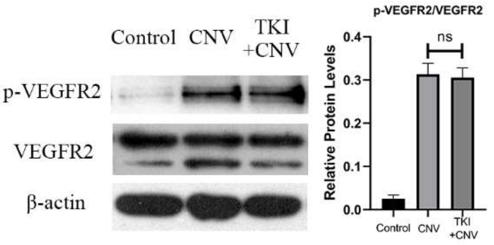

Supplementary Figure 3. VEGFR2 protein phosphorylation in mice choroid. CNV was induced in untreated C57/BL6 mice (CNV group) and TKI intravitreal injection mice (TKI + CNV group). Choroid protein samples were extracted 1week after laser injury. Mice without laser treatment were set as control. No significant difference was found between CNV group and TKI + CNV group. The mean gray value of the Western blot was determined and described as mean ± SEM. n =10 for each of the 3 groups. Representative results were obtained from three independent experiments.

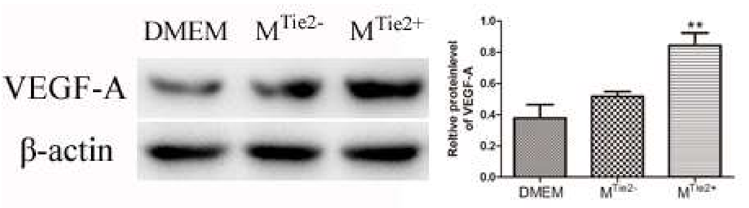

Supplementary Figure 4. The relative VEGF-A protein expression of b.End3 under different macrophage supernatant. The b.End3 cells were incubated with DMED medium (DMEM), macrophage supernatant from the control mice (M^{Tie2+}), and macrophage supernatant from the TEM-KO mice (M^{Tie2-}) for 24 h, respectively. Representative results, as determined by Western blot, from three independent experiments are presented. Values represent mean ± SEM.

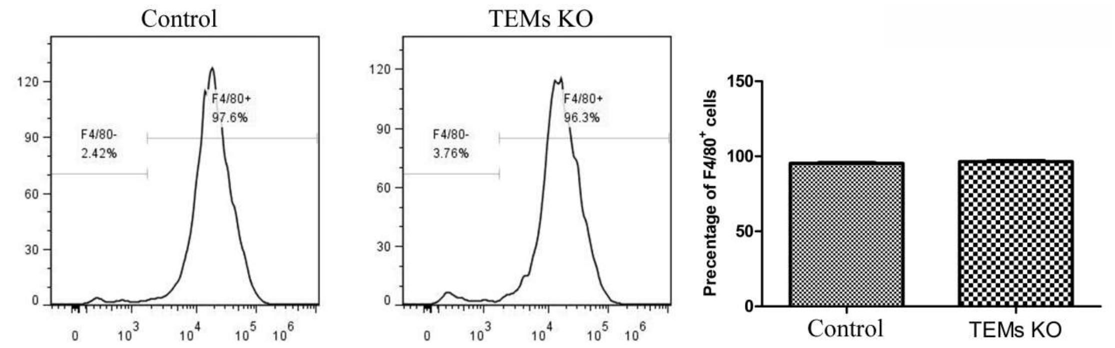

Supplementary Figure 5. FACS analysis for percentage of F4/80^{+} cells in murine peritoneal macrophages. Macrophages were harvested from control and TEM-KO mcie, respectively. No significant difference was observed between the two groups.

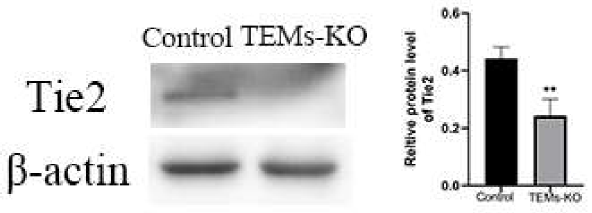

Supplementary Figure 6. Western blot analysis for Tie2 expression on macrophages. Protein samples were extracted from control and TEM-KO mice peritoneal macrophages, respectively. Rarely Tie2 expression was detected on TEM-KO group.

Supplementary Table1. The relative gene expression levels were calculated using the 2−ΔΔCt method.

Random primers were shown as following:

Gene names	primer sequences
IL-1α	Forword: 5’-CGAAGACTACAGTTCTGCCATT-3’
	Reverse: 5’-GACGTTTCAGAGGTTCTCAGAG-3’
IL-10	Forword: 5’-CAGCCGGGAAGACAATAACTG-3’
	Reverse: 5’-CCGCAGCTCTAGGAGCATGT-3’
VEGF-A	Forword: 5’- AGGGAAGAGGAGGAGATGAGA-3’
	Reverse: 5’- GGCTGGGTTTGTCGGTGTT-3’
bFGF	Forword: 5’- AAGAGCGACCCACACGTCAAAC -3’
	Reverse: 5’- GTAACACACTTAGAAGCCAGCAGCC -3’

Pictures of full-length gels and blots

Figure 3b

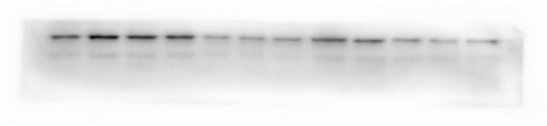

p-AKT

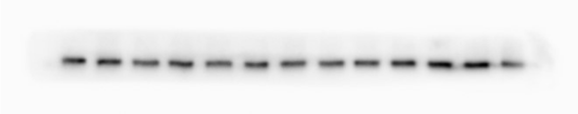

AKT

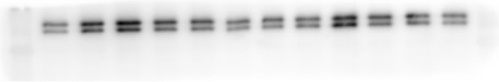

p-ERK

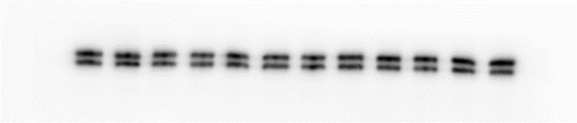

ERK

VEGF-A

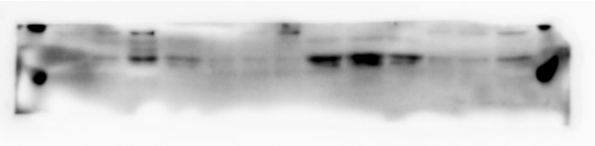

Cleaved-caspase3

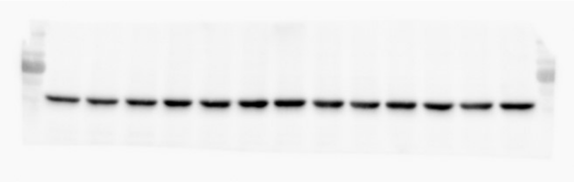

Actin

Figure 5d

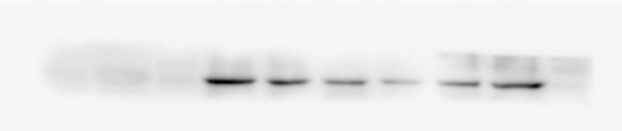

p-AKT

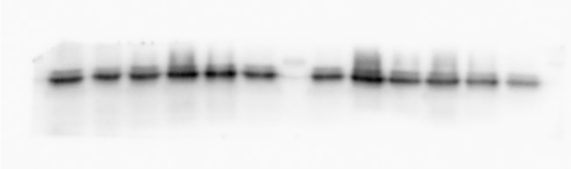

AKT

p-ERK

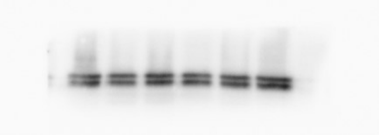

ERK

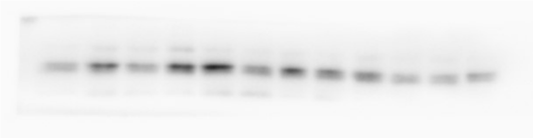

VEGF-A

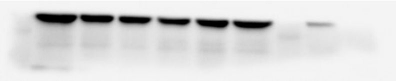

Actin

Figure5f

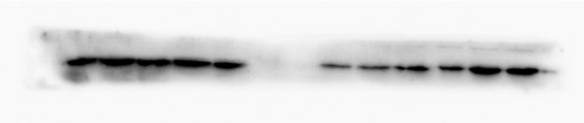

cleaved-caspase3

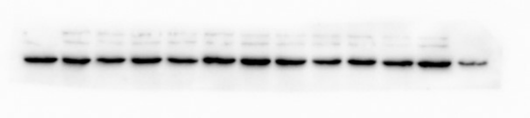

Actin

Supplementary Figure 3

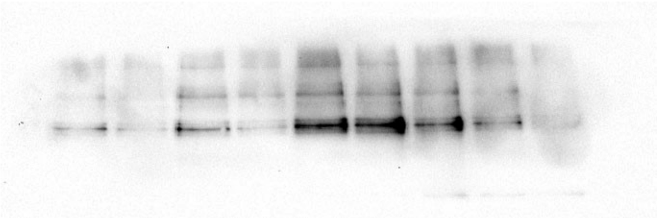

pVEGFR2

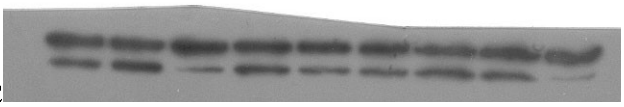

VEGFR2

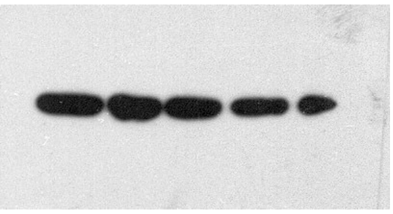

Actin

Supplementary Figure 4

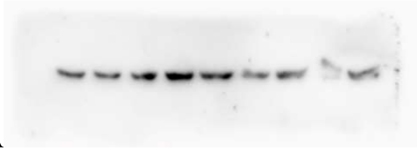

VEGF-A

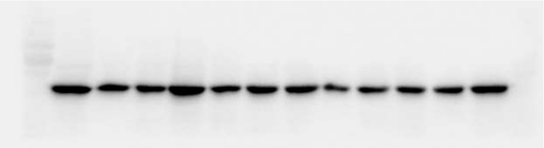

Actin

Supplementary Figure 5

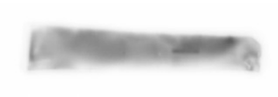

Tie2

Actin
